# Supplementary figures and images for: Genome rearrangements and selection in multi-chromosome bacteria Burkholderia spp
Source: BMC Genomics. 2018 Dec 27;19:965. doi: 10.1186/s12864-018-5245-1 (PMC6307245; doi:10.1186/s12864-018-5245-1)

A

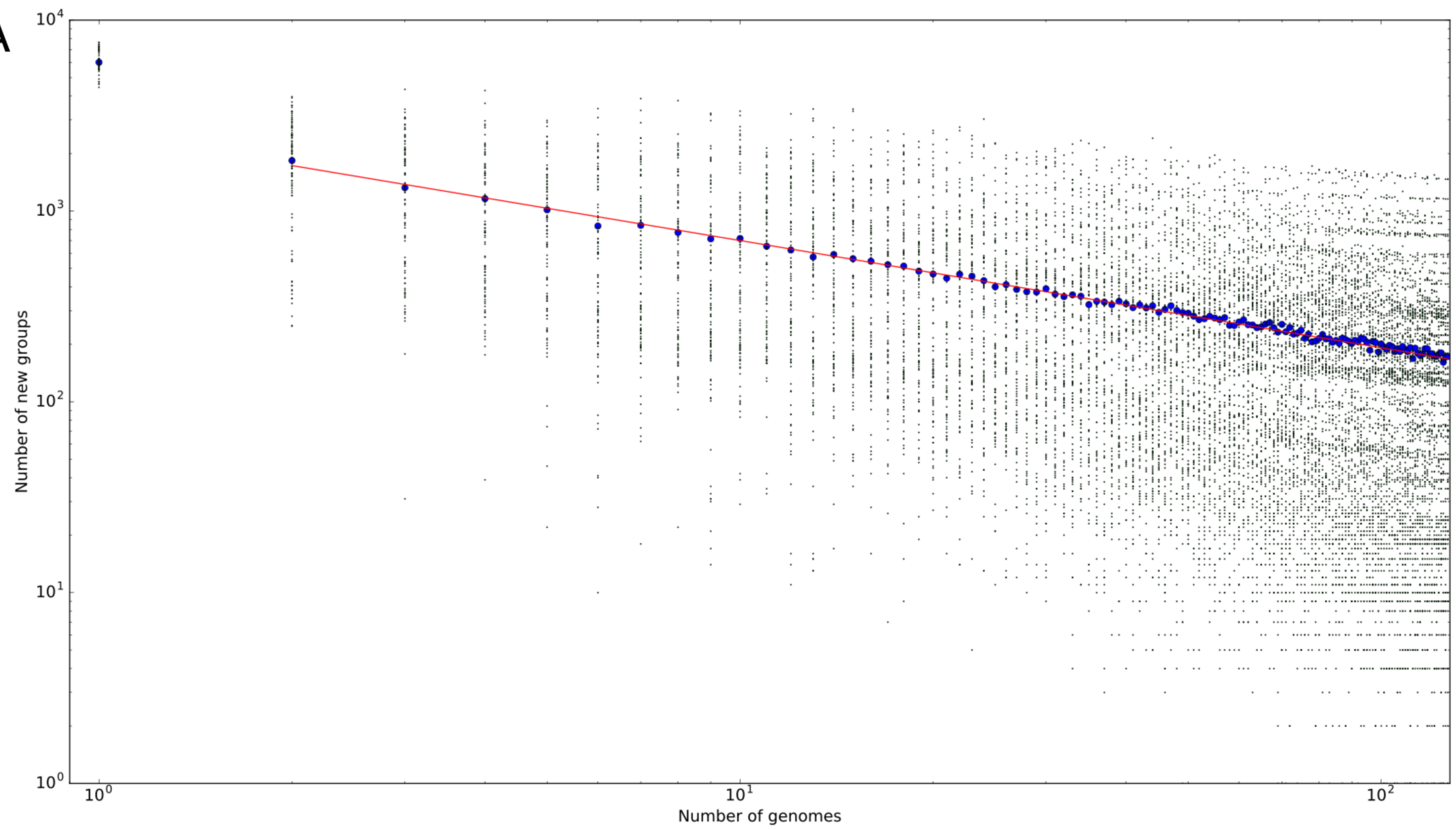

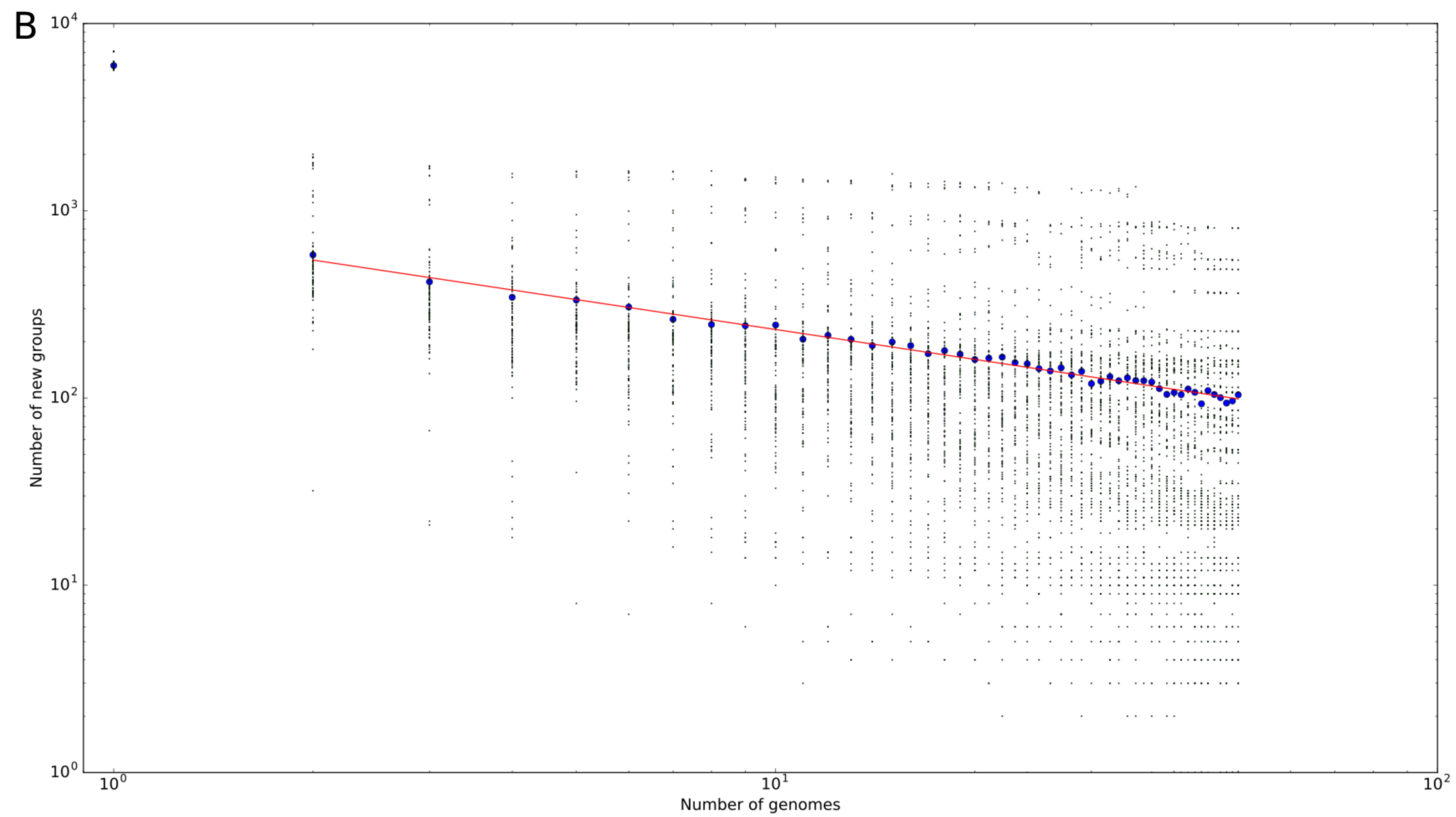

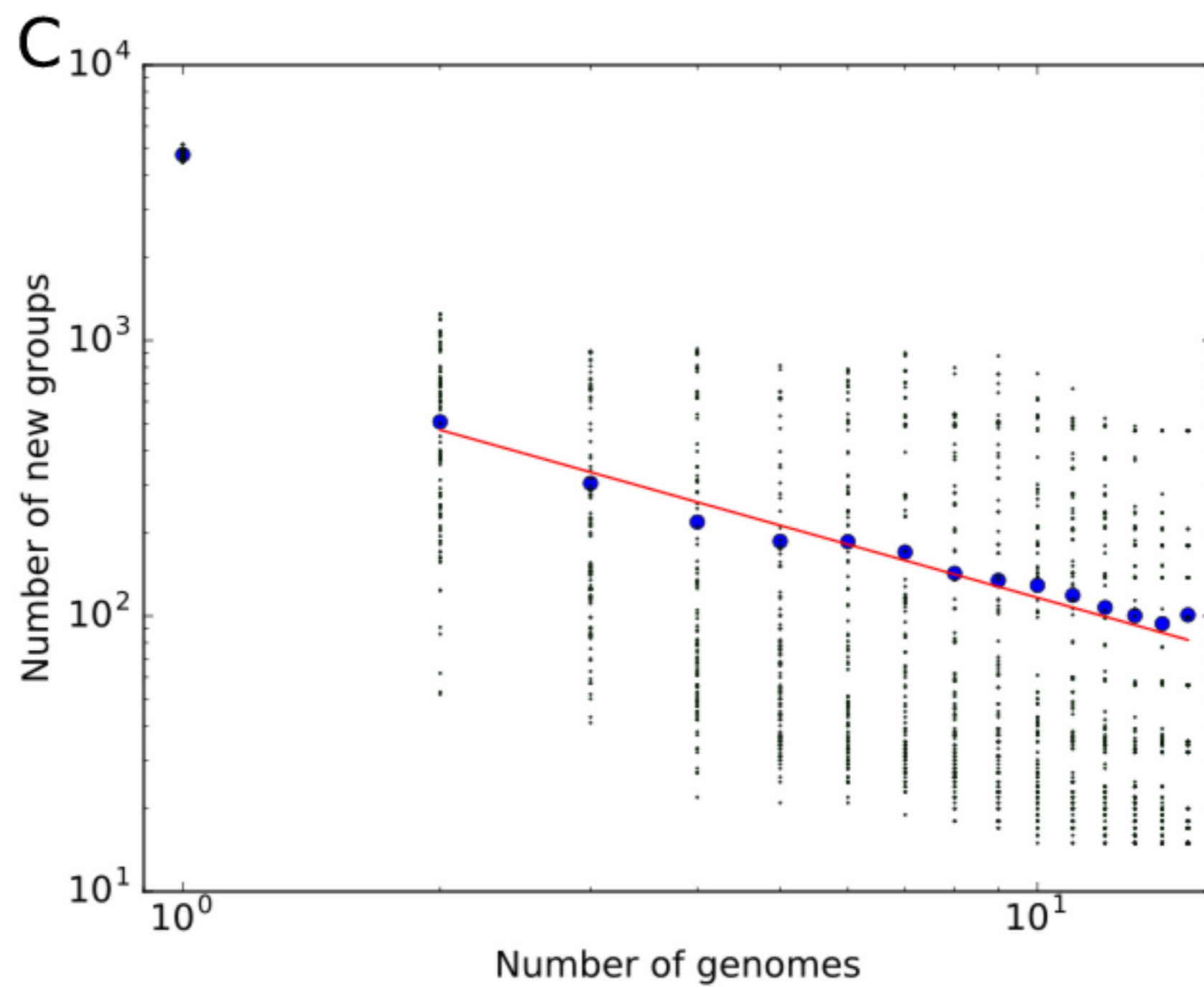

Supplement: Supplementary file 1 — Figure S1. The number of new genes added to the pangenome upon addition of new strains. (a) Burkholderia spp., (b) B. pseudomallei, and (c) B. mallei. The number of new genes is plotted as a function of the number (n) of strains sequentially added (see the model in [81]). For each n, points are the values obtained for different strain combinations; red symbols are the averages of these values. The superimposed line is a fit with a decaying power law y y=A∗nB. (PDF 1224 kb) [file 12864_2018_5245_MOESM1_ESM.pdf]

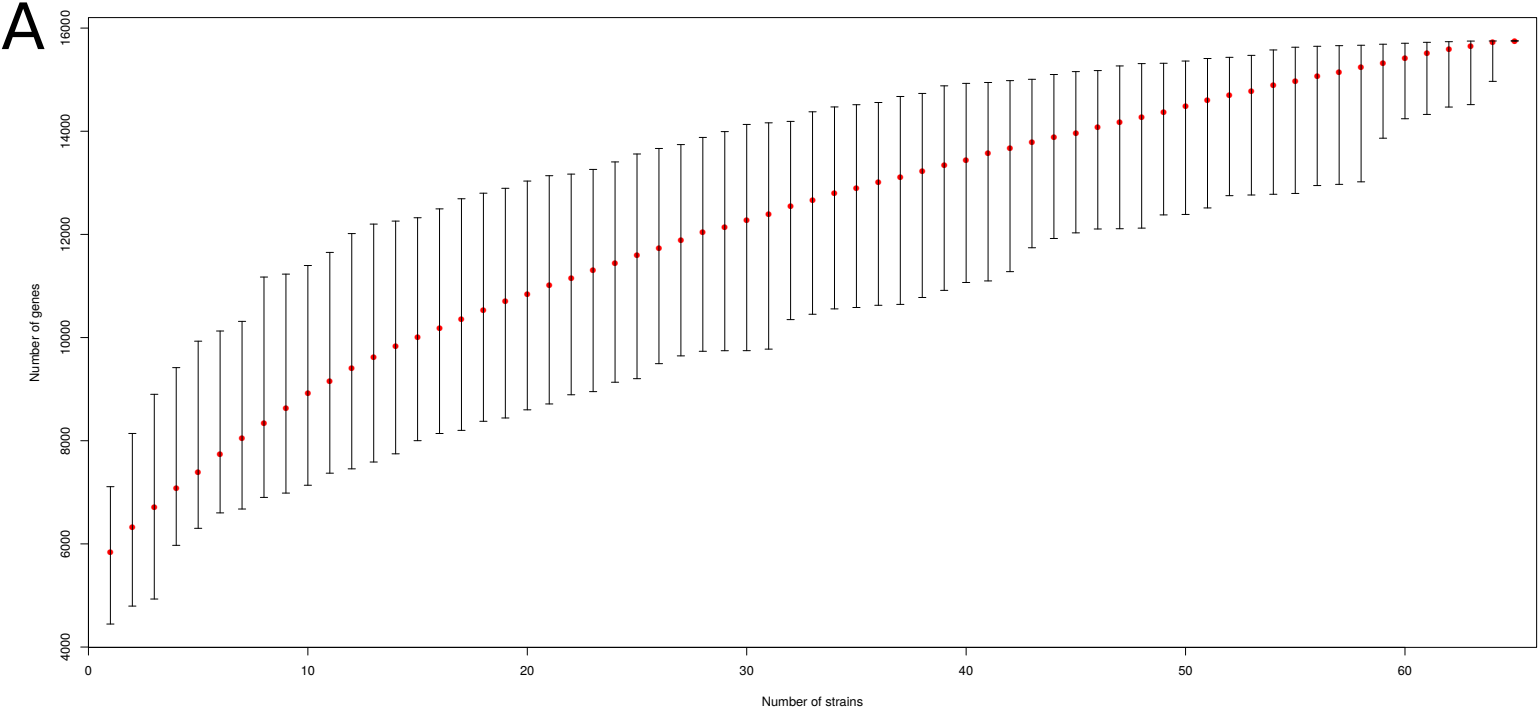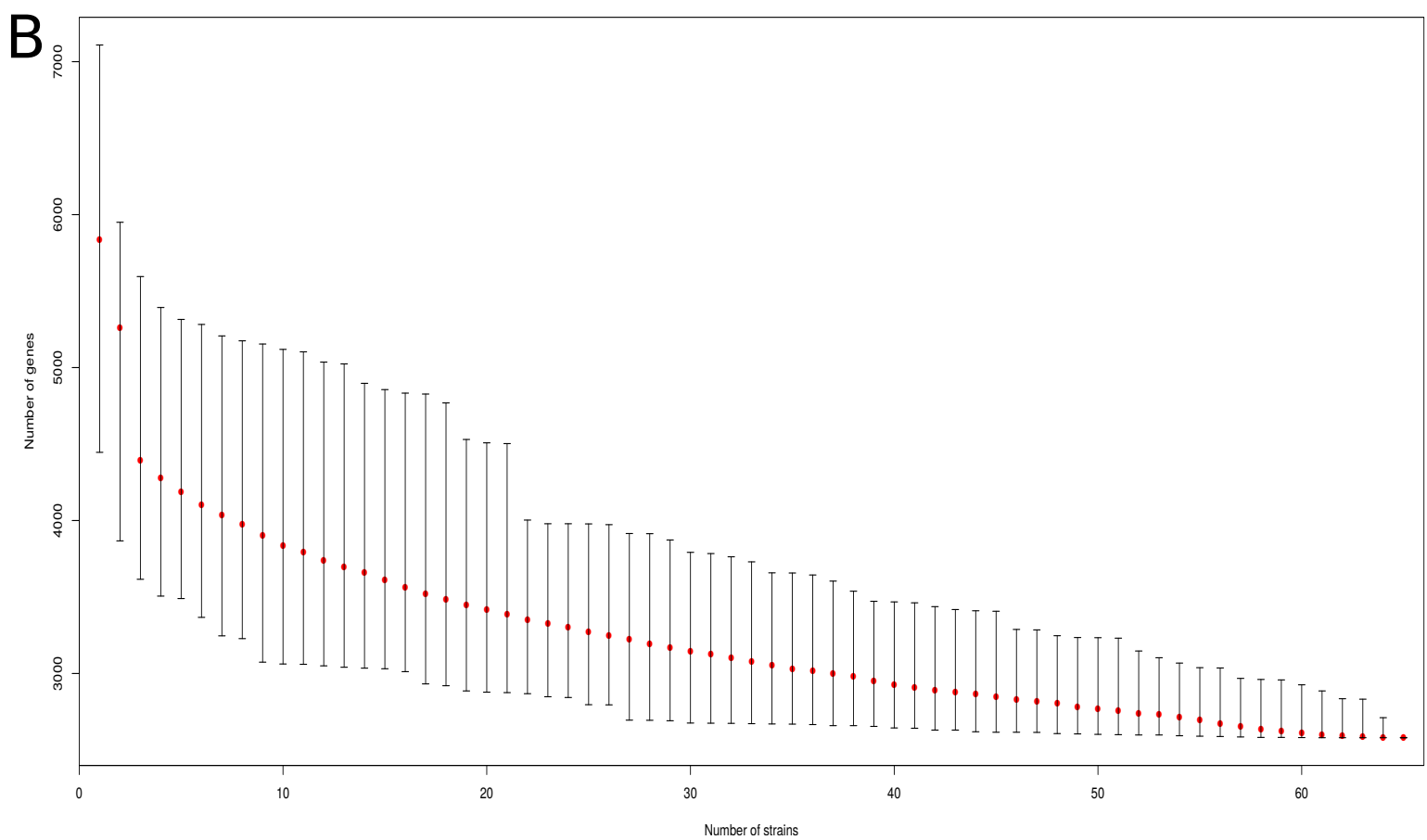

Supplement: Supplementary file 2 — Figure S2. Pan-genome (a) and core-genome (b) size of B. pseudomallei strains. (PDF 21 kb) [file 12864_2018_5245_MOESM2_ESM.pdf]

**A**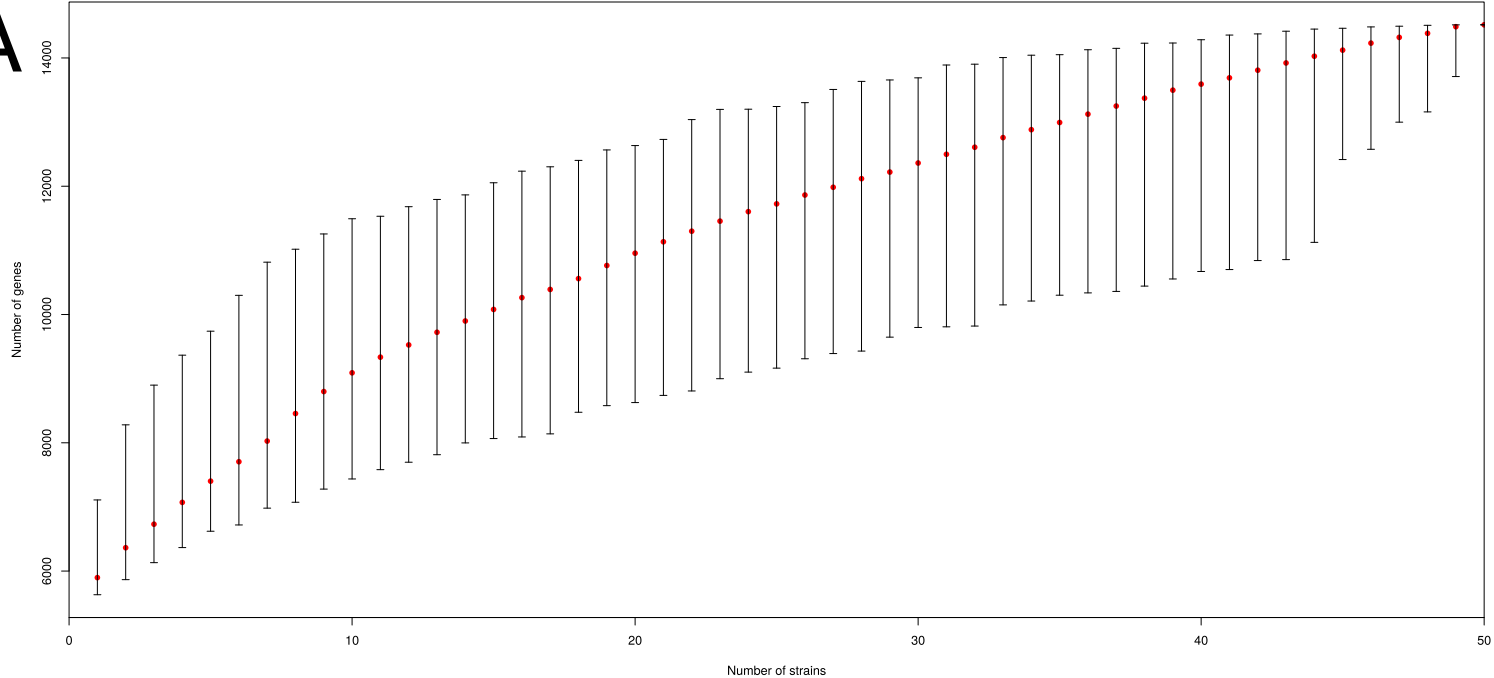**B**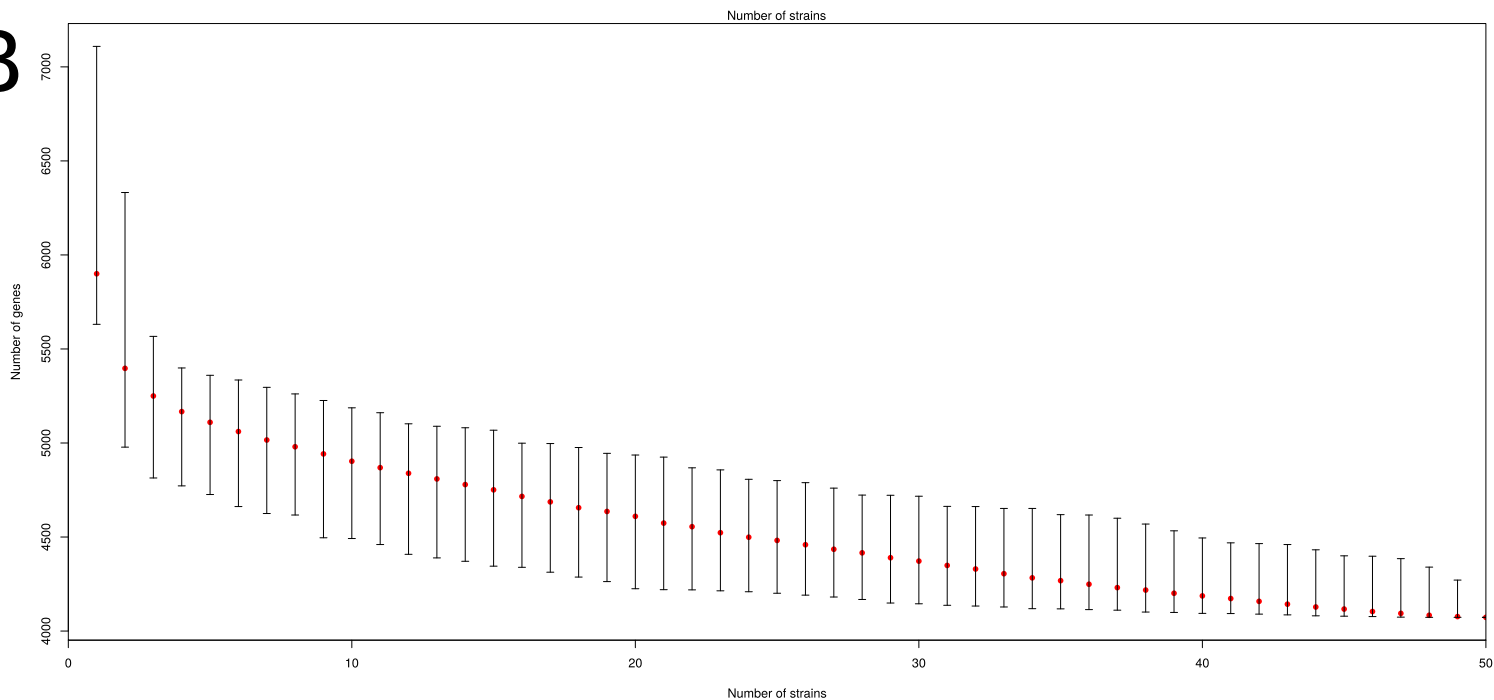

Supplement: Supplementary file 3 — Figure S3. Pan-genome (a) and core-genome (b) size of B. mallei strains. (PDF 31 kb) [file 12864_2018_5245_MOESM3_ESM.pdf]

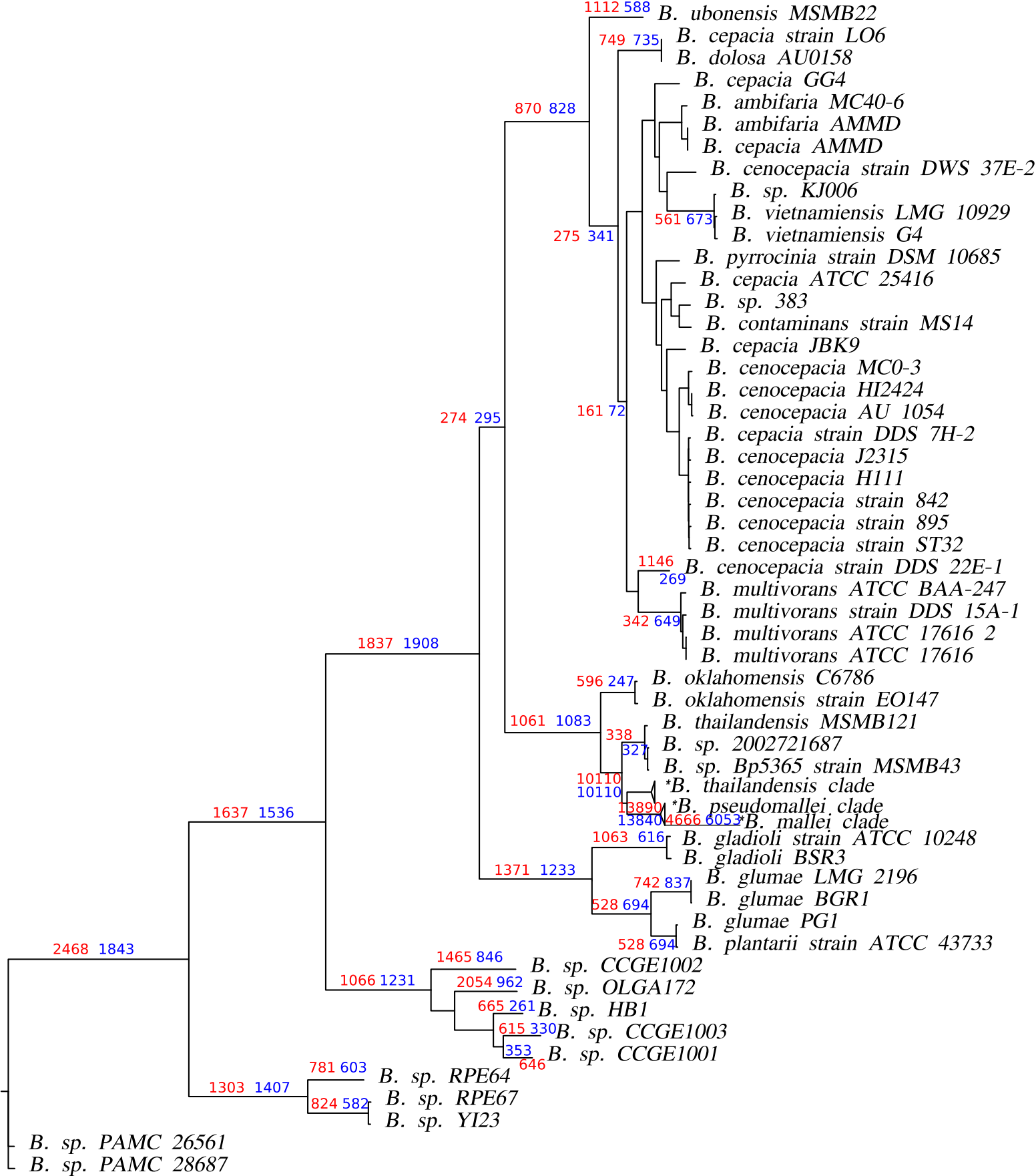

Supplement: Supplementary file 4 — Figure S4. Gene flow during Burkholderia evolution. Red and blue numbers are, respectively, the numbers of gained and lost genes on a given branch. (PDF 414 kb) [file 12864_2018_5245_MOESM4_ESM.pdf]

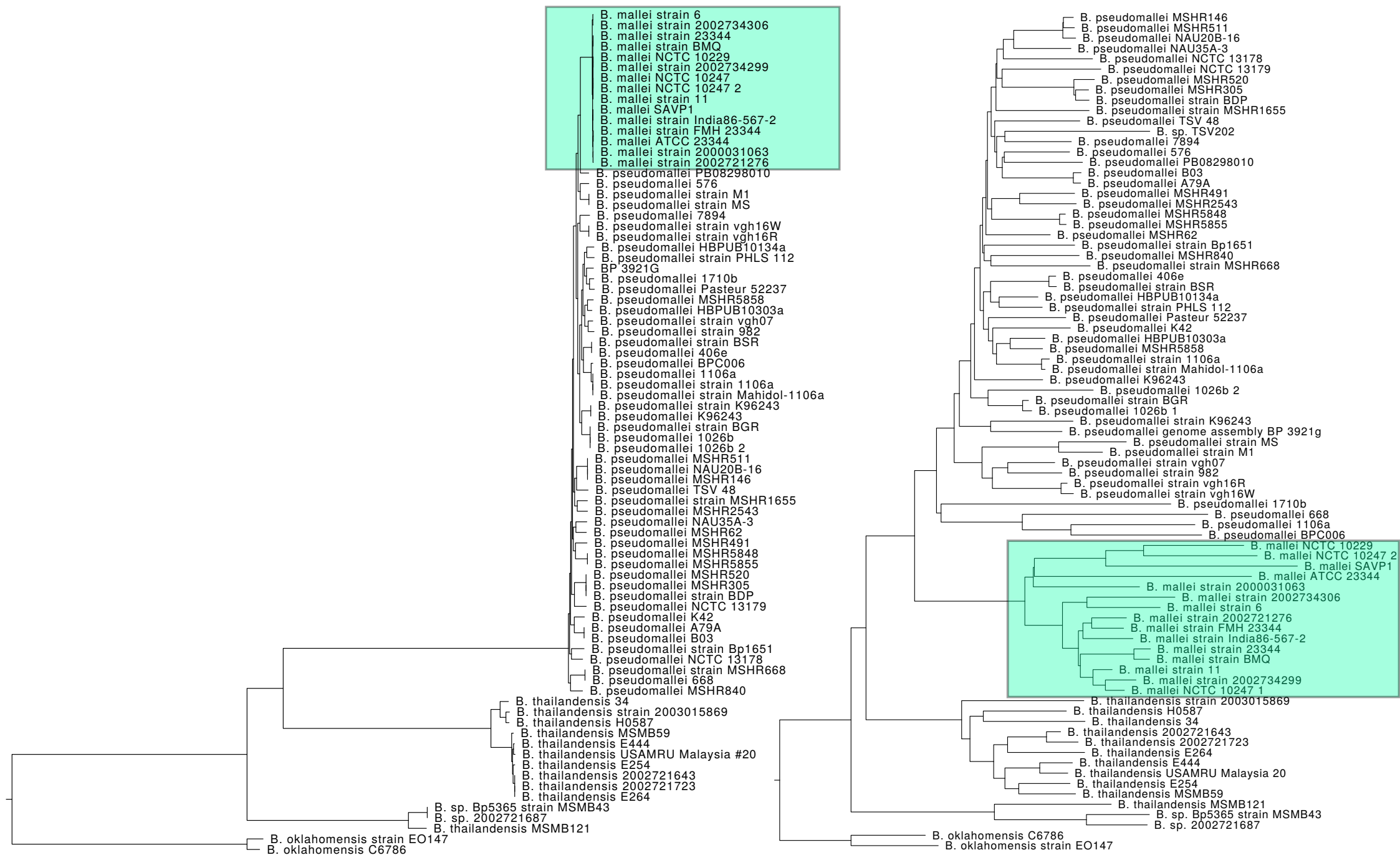

Supplement: Supplementary file 5 — Figure S5. Comparison the topologies of phylogenetic trees based on the protein sequence similarity of single-copy universal genes and the gene content. (PDF 26 kb) [file 12864_2018_5245_MOESM5_ESM.pdf]

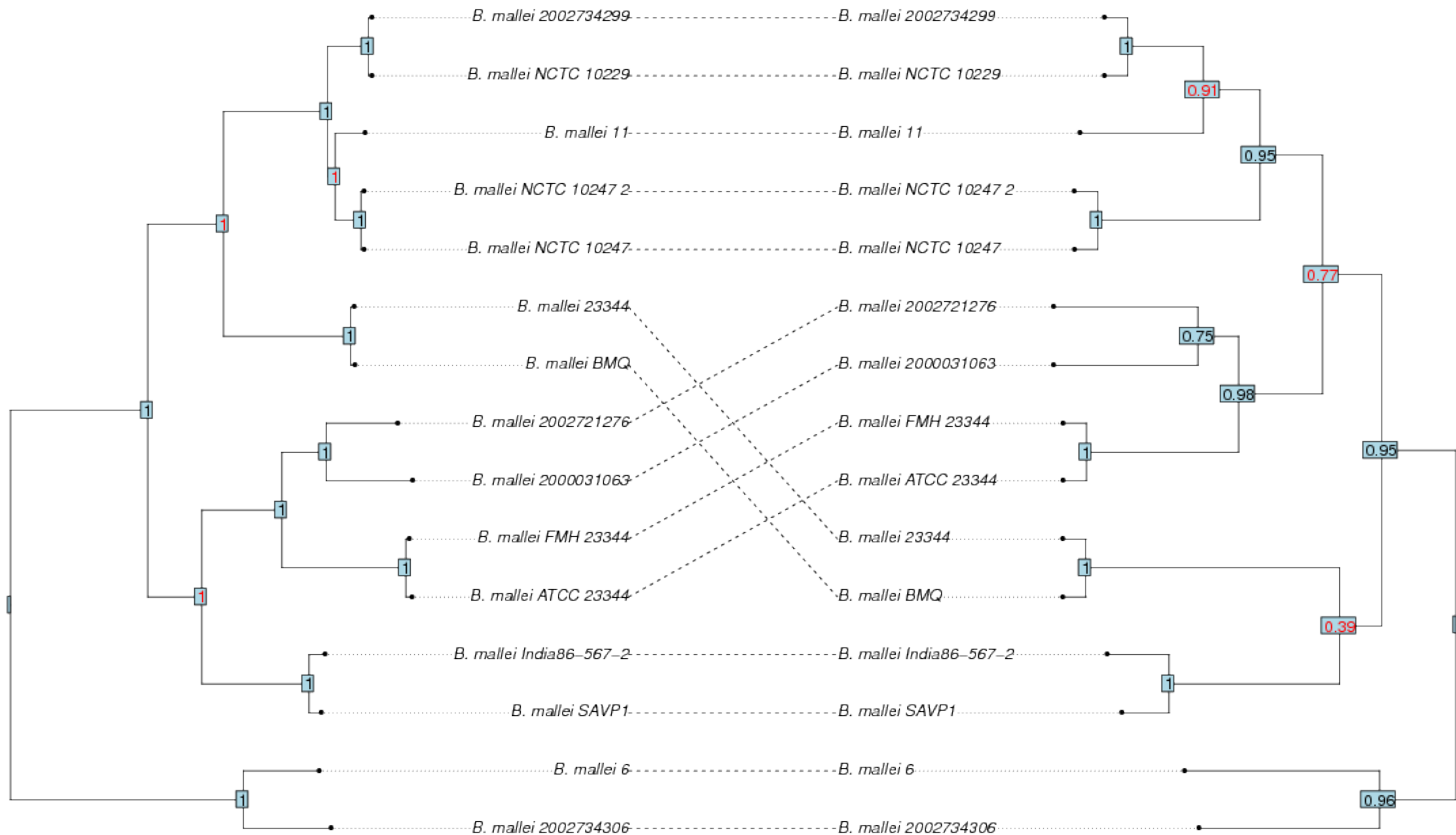

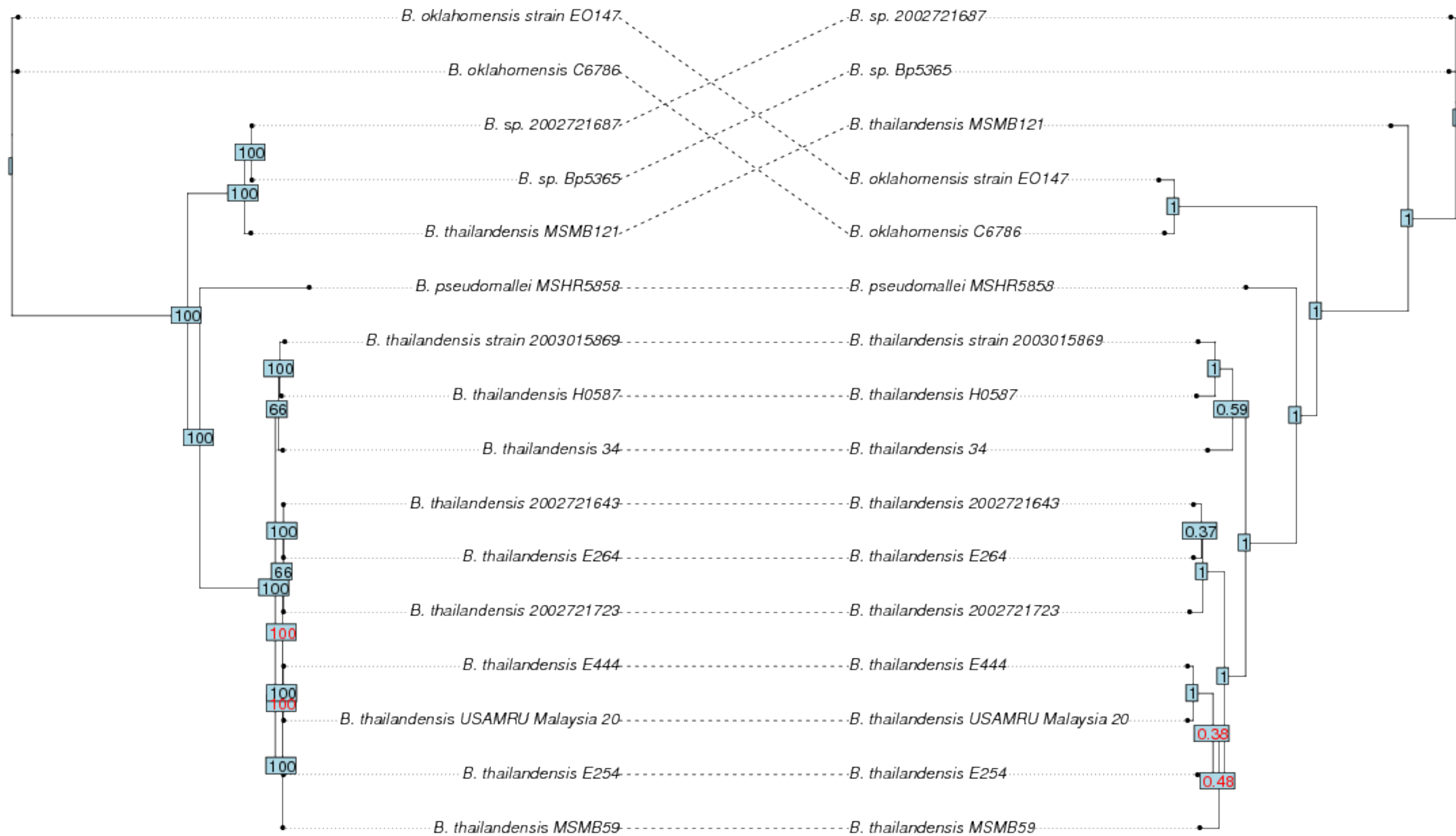

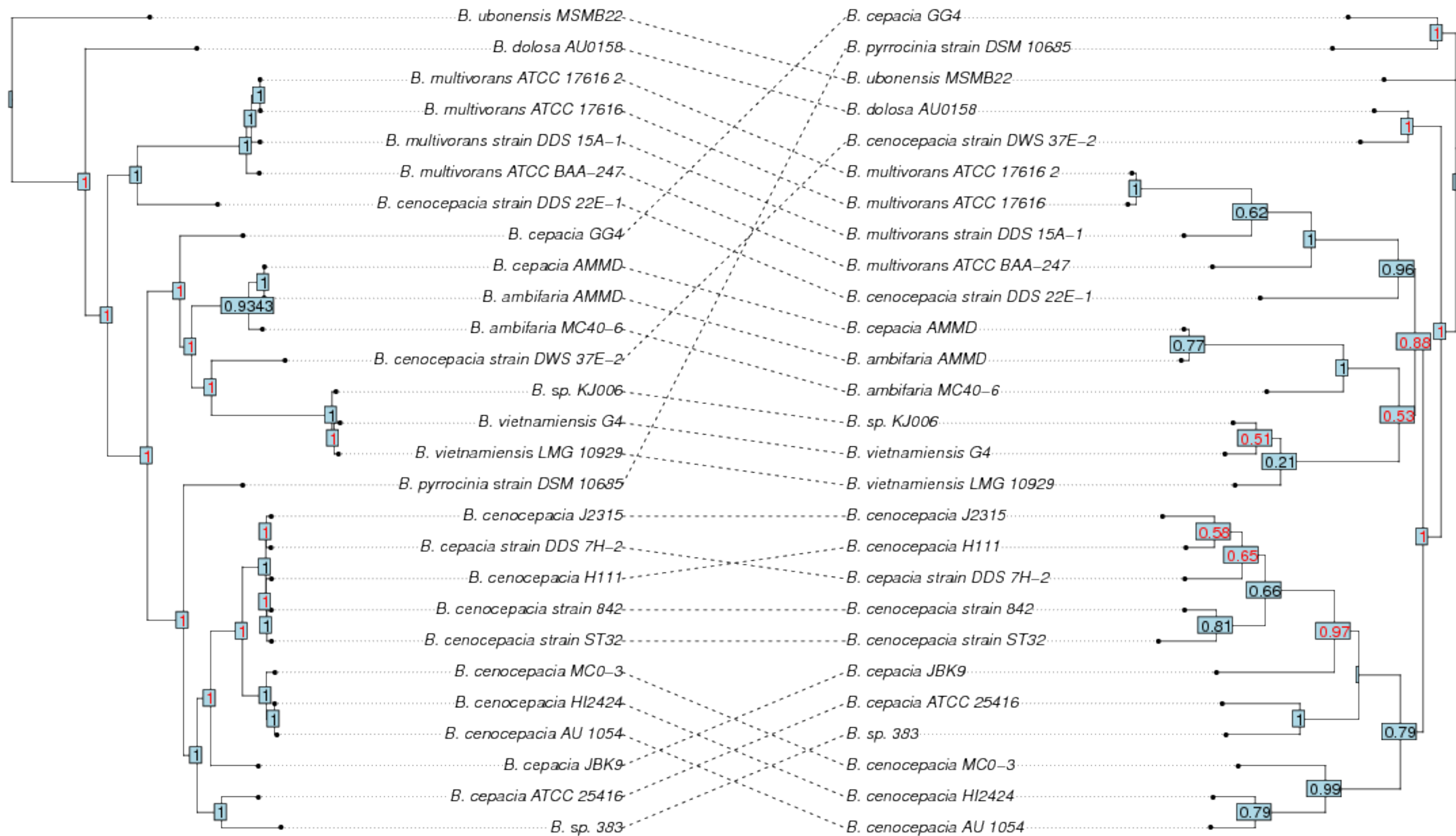

Supplement: Supplementary file 7 — Figure S7. Tanglegrams showing the differences between the tree topologies based on the protein sequence similarity of single-copy universal genes and the tree topologies based on the synteny blocks arrangements. (a) B. mallei clade; (b) B. thailandensis clade; (c) B. cepacia group. (PDF 524 kb) [file 12864_2018_5245_MOESM7_ESM.pdf]

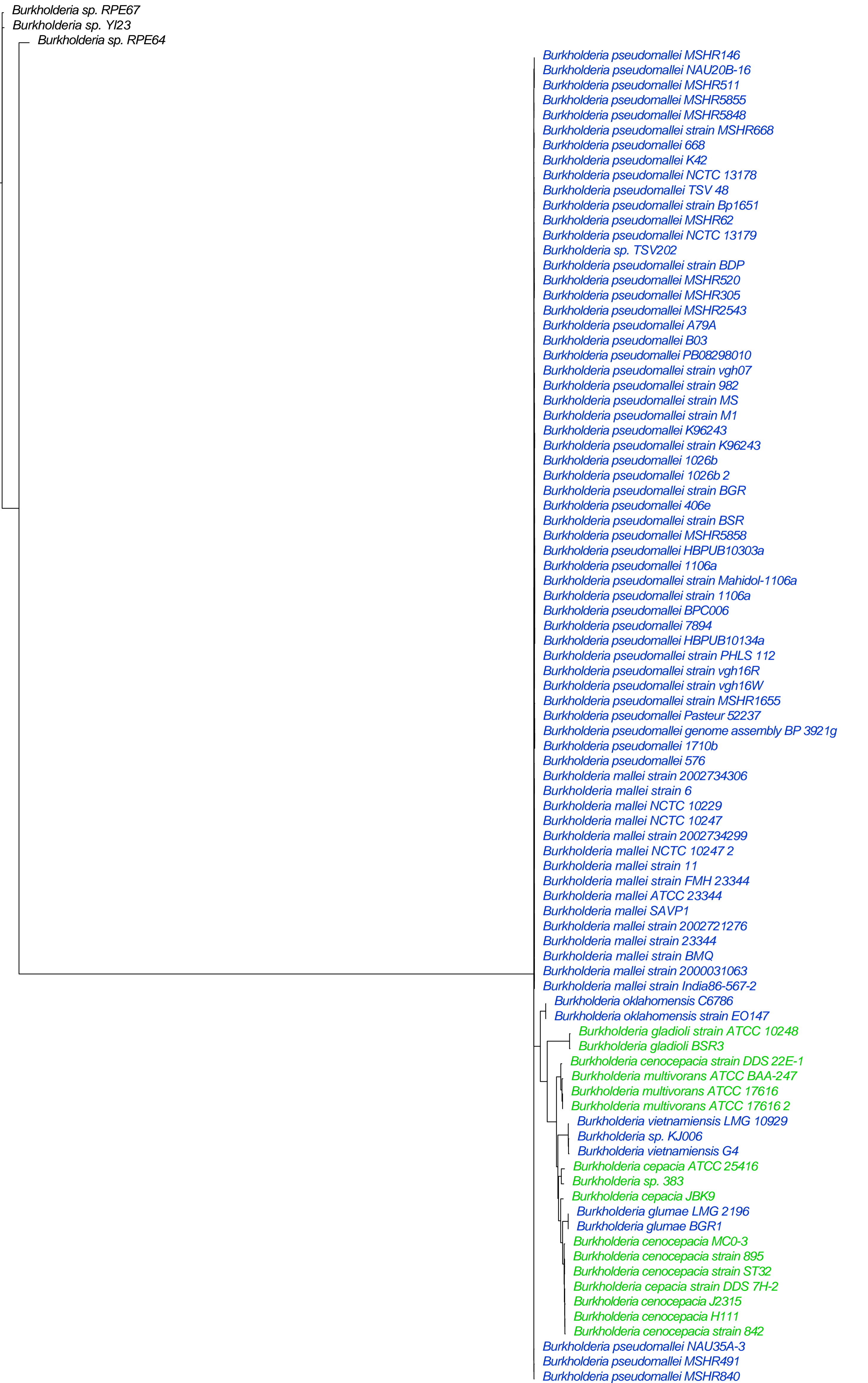

Supplement: Supplementary file 8 — Figure S8. Phylogenetic tree constructed based on the concatenation of alignments of genes forming the genomic island. (PDF 6 kb) [file 12864_2018_5245_MOESM8_ESM.pdf]

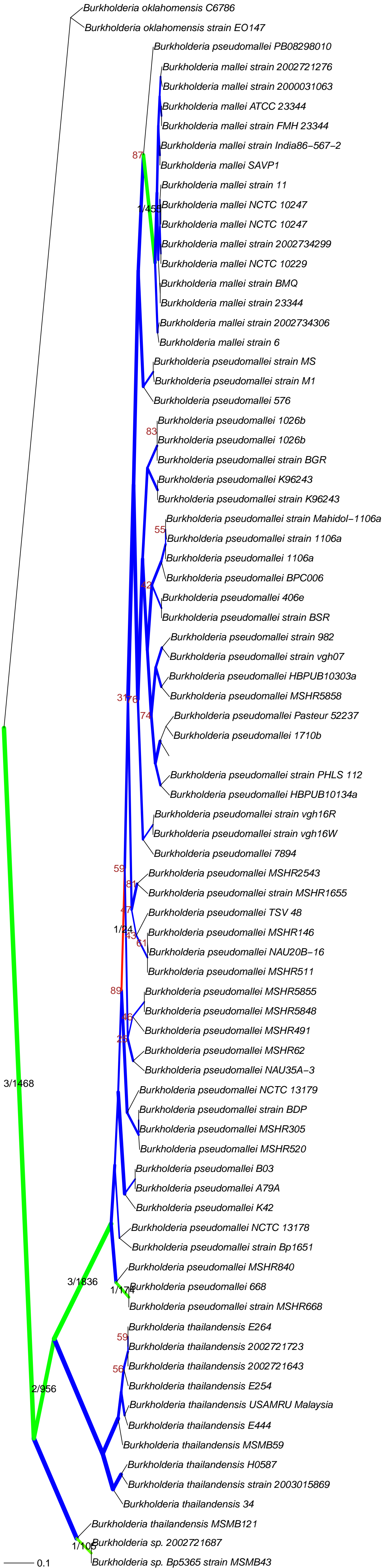

Supplement: Supplementary file 9 — Figure S9. Phylogenetic tree showing detected events of positive selection. (PDF 8 kb) [file 12864_2018_5245_MOESM9_ESM.pdf]
