# Supplementary material for: Genome rearrangements and selection in multi-chromosome bacteria Burkholderia spp
Source: BMC Genomics. 2018 Dec 27;19:965. doi: 10.1186/s12864-018-5245-1 (PMC6307245; doi:10.1186/s12864-018-5245-1)
Supplement: Supplementary file 6 — Figure S6. Whole-genome alignments of cepacia strains that were not included in the rearrangement analysis due to likely artifacts of the genome assembly. (a) Burkholderia sp. 383 and B. cepacia strain LO6 (b) Burkholderia sp. 383 and B. contaminans strain MS14, (c) Burkholderia sp. 383 and B. cenocepacia strain 895, (d) B. cepacia strain LO6 and B. cenocepacia strain 895. (PDF 25,604 kb) [file 12864_2018_5245_MOESM6_ESM.pdf]

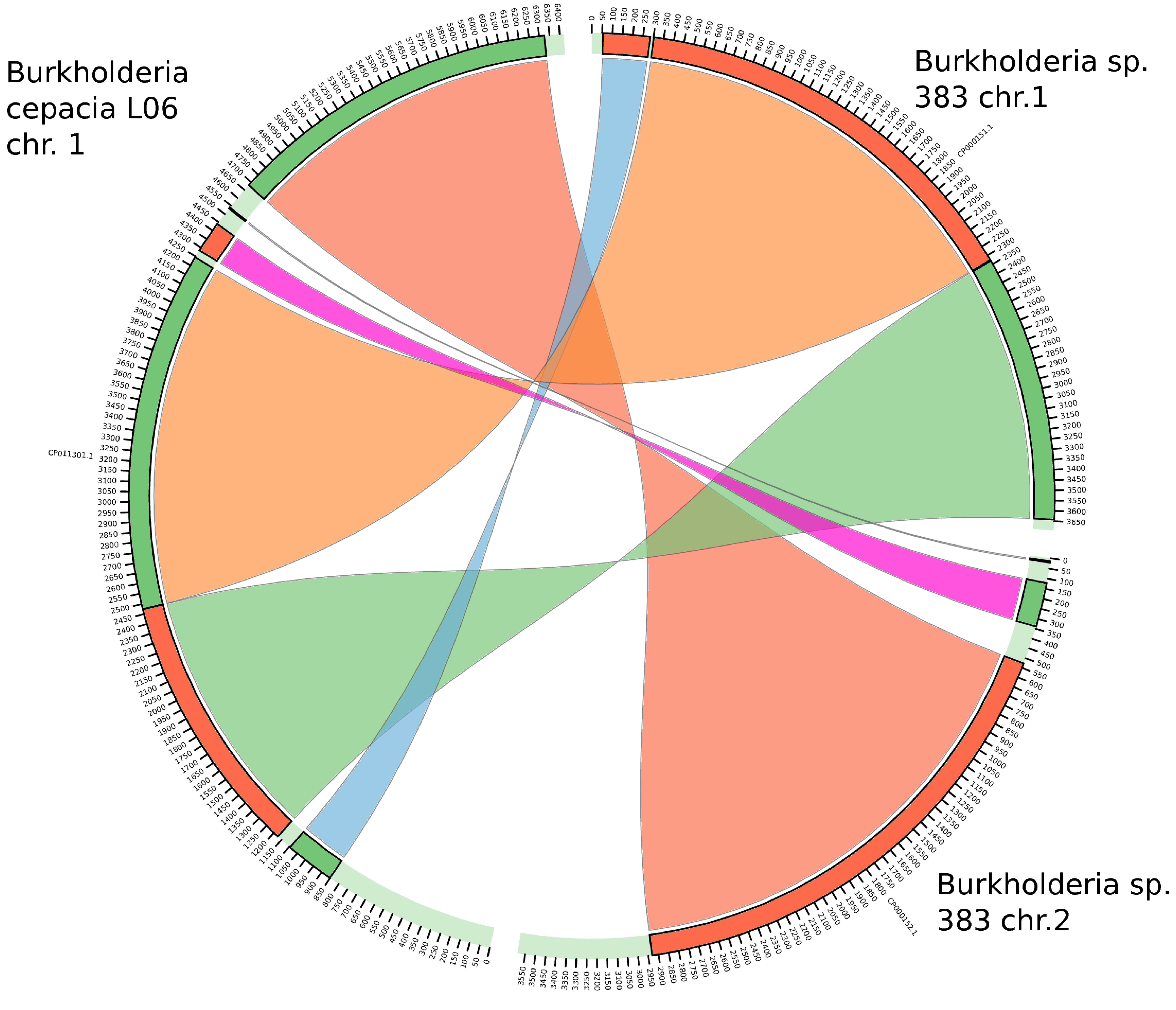
Burkholderia cepacia L06 chr. 1
Burkholderia sp. 383 chr.1
Burkholderia sp. 383 chr.2
CP011301.1
CP000151.1
CP000152.1

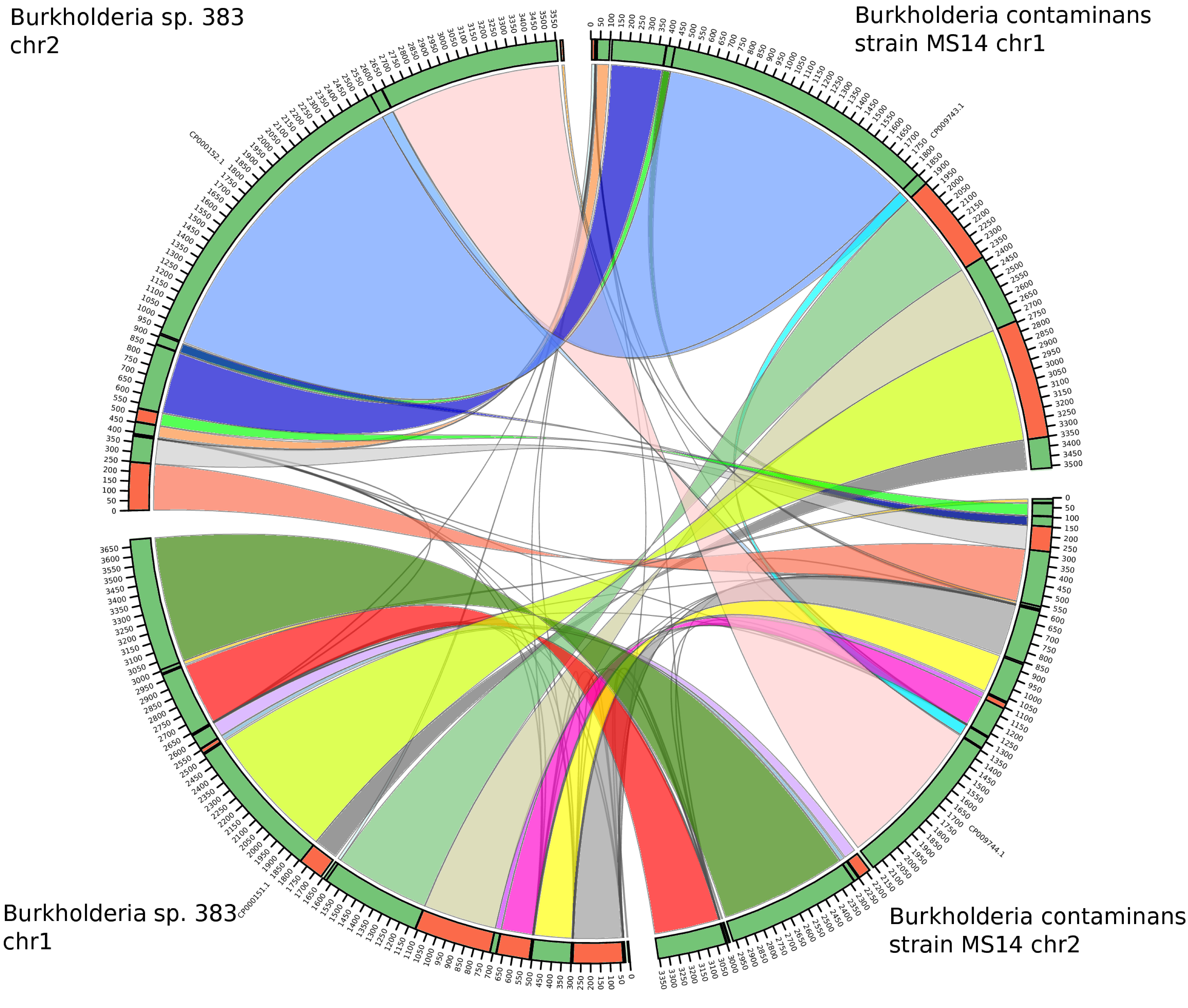
Burkholderia sp. 383
chr2
Burkholderia contaminans
strain MS14 chr1
Burkholderia sp. 383
chr1
Burkholderia contaminans
strain MS14 chr2
CP000152.1
CP009743.1
CP000151.1
CP009744.1

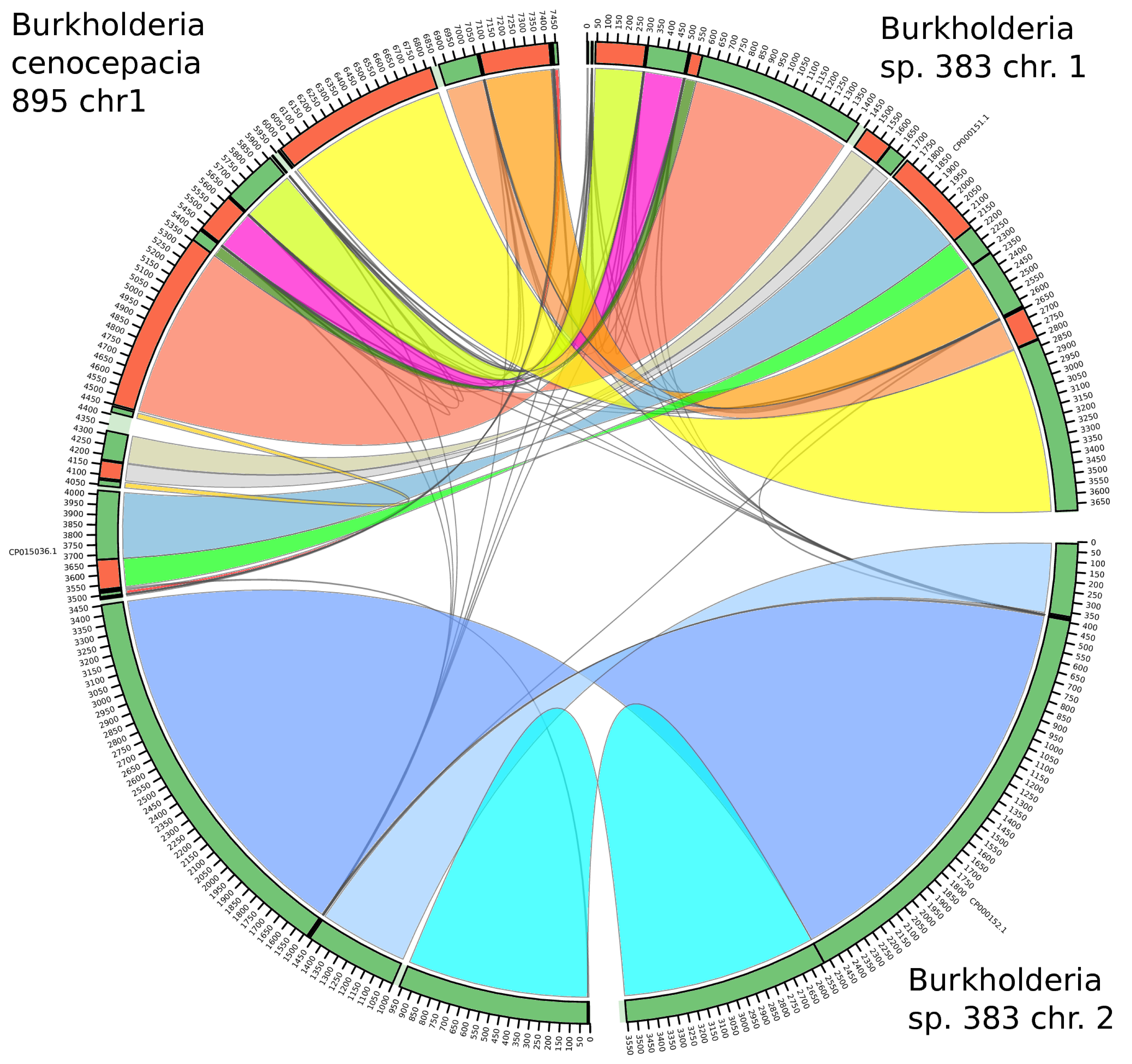
Burkholderia cenocepacia 895 chr1
Burkholderia sp. 383 chr. 1
Burkholderia sp. 383 chr. 2
CP015036.1
CP000151.1
CP000152.1

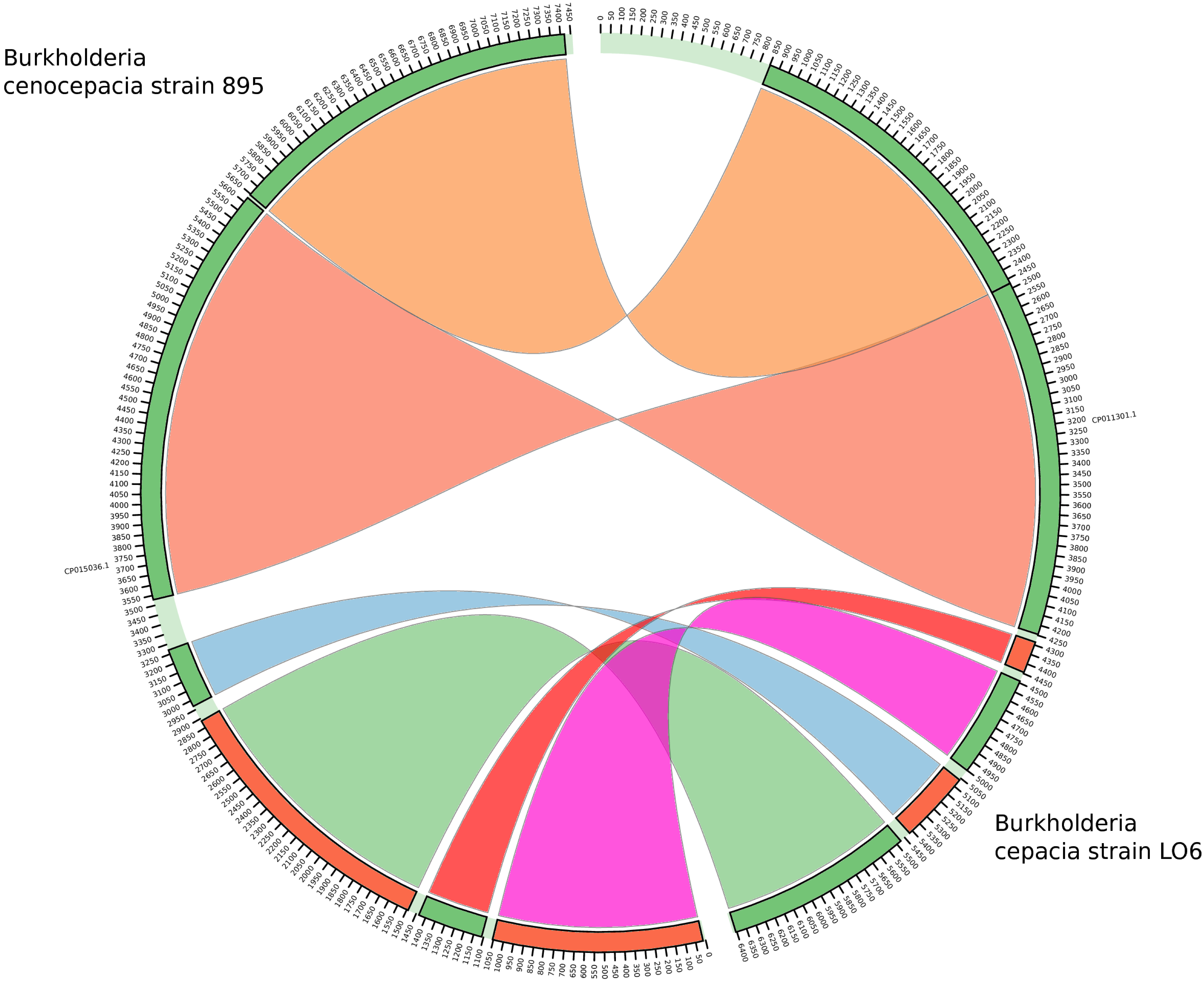
Burkholderia
cenocepacia strain 895
Burkholderia
cepacia strain LO6
CP011301.1
CP015036.1
