# Supplementary material for: Genome rearrangements and selection in multi-chromosome bacteria Burkholderia spp
Source: BMC Genomics. 2018 Dec 27;19:965. doi: 10.1186/s12864-018-5245-1 (PMC6307245; doi:10.1186/s12864-018-5245-1)
Supplement: Supplementary file 12 — Table S3. Linear models (a) of average ω (negative selection, estimated using M8); (b) expression level [35]. (PDF 92 kb) [file 12864_2018_5245_MOESM12_ESM.pdf]

Table 1: Linear models

A) Linear model of purifying selection strength: ω_0; smaller values of ω_0 indicate stronger purifying selection. Adjusted R^2: 0.2976, p-value $< 2.2 \cdot 10^{-16}$. Model formula: ω_0 ~ `gene length + distance from the origin of replication + gene placement on the leading strand + gene placement in the first chromosome + average expression level + average GC content + GC content standard deviation + sum of branch lengths`.

	Estimate	Std. Error	t value	p-value
(Intercept)	0.060	0.064	0.943	0.3463
gene length	-0.102	0.042	-2.413	0.0162
distance from the origin of replication	0.001	0.041	0.032	0.9742
gene placement on the leading strand	0.059	0.065	0.896	0.3707
gene placement in the first chromosome	-0.096	0.076	-1.267	0.2057
average expression level	-0.075	0.033	-2.247	0.0250
average GC content	0.052	0.041	1.284	0.1998
GC content standard deviation	0.059	0.041	1.430	0.1532
sum of branch lengths	0.412	0.039	10.578	$< 2 \cdot 10^{-16}$

B) Linear model of average expression level. Adjusted R^2: 0.1859, p-value $< 2.2 \cdot 10^{-16}$. Model formula: `average expression level ~ gene length + gene placement in the first chromosome + distance from the origin of replication + gene placement on the leading strand + average GC content + LRT statistic value +` ω_0 `+ sum of branch lengths`.

	Estimate.str	Std. Error	t value	p.value
(Intercept)	-0.310	0.084	-3.682	0.000256
gene length	0.029	0.047	0.623	0.533667
gene placement in the first chromosome	0.505	0.099	5.102	$4.75 \cdot 10^{-7}$
distance from the origin of replication	-0.069	0.055	-1.258	0.208840
gene placement on the leading strand	-0.016	0.087	-0.189	0.850215
average GC content	-0.291	0.052	-5.553	$4.54 \cdot 10^{-8}$
LRT statistic	-0.013	0.046	-0.282	0.778420
ω_0	-0.134	0.060	-2.249	0.024969
sum of branch lengths	-0.111	0.056	-1.959	0.050658
